# Ethical and bioethical issues in physical therapy: A systematic scoping review

**DOI:** 10.1093/ptj/pzag011

**Published:** 2026-02-02

**Authors:** Gianluca Bertoni, Sara Patuzzo Manzati, Federica Pagani, Marco Testa, Simone Battista

**Affiliations:** Department of Neurosciences, Rehabilitation, Ophthalmology, Genetics, Maternal and Child Health, University of Genoa, Campus of Savona, Savona, Italy; Department of Clinical and Experimental Sciences, University of Brescia, Brescia, Italy; Training Unit, Azienda Sociosanitaria Territoriale di Cremona, Cremona, Italy; Department of Surgery, Dentistry, Paediatrics and Gynaecology, University of Verona, Verona, Italy; Department of Neurosciences, Rehabilitation, Ophthalmology, Genetics, Maternal and Child Health, University of Genoa, Campus of Savona, Savona, Italy; Department of Neurosciences, Rehabilitation, Ophthalmology, Genetics, Maternal and Child Health, University of Genoa, Campus of Savona, Savona, Italy; School of Health & Society, Centre for Human Movement and Rehabilitation, University of Salford, Salford, Greater Manchester, UK

**Keywords:** Ethical, Ethics, Literature, Physical therapy, Profession, Reasoning

## Abstract

**Importance:**

Ethical and bioethical issues are central to the identity and practice of physical therapy. A comprehensive overview of how these issues are addressed in the literature is essential for advancing education, clinical practice, and professional reflection.

**Objective:**

The objective was to systematically map ethical and bioethical issues in the physical therapy literature, describe the methodologies employed, and identify key gaps to inform education, practice, and policy.

**Data Sources:**

Medline (via PubMed), Embase, Cochrane Central, CINAHL, PsycINFO, PEDro, grey literature sources, and academic library resources were searched from inception to October 2024. The review protocol was prospectively published on medRxiv.

**Study Selection:**

Studies addressing ethical or bioethical issues in physical therapy were included, encompassing both normative and descriptive (empirical) approaches. After screening titles, abstracts, and full texts, 108 studies met the inclusion criteria.

**Data Extraction and Synthesis:**

Data were extracted using a modified Joanna Briggs Institute standardized form. A narrative synthesis was conducted to map ethical themes and characterize methodological approaches across studies.

**Main Outcomes and Measures:**

Identification and mapping of ethical and bioethical themes and characterization of research methodologies applied.

**Results:**

A total of 15,464 records were identified; 3223 duplicates were removed. Of 12,241 titles and abstracts screened, 385 full texts were assessed, and 108 studies were included. Major themes included ethical reasoning (*n* = 33), ethical reasoning and education (*n* = 19), ethical theories (*n* = 12), care relationships (*n* = 15), justice and equity (*n* = 8), perception of ethical issues (*n* = 13), and codes of ethics (*n* = 8). Key challenges involved physical touch, informed consent, professional boundaries, and moral distress. Structural barriers, cultural contexts, and disparities in ethics education were recurring concerns. Ethical reasoning was often situational and intuitive, whereas formal codes were frequently perceived as disconnected from clinical practice.

**Conclusions and Relevance:**

Ethical complexities in physical therapy arise from its embodied, relational, and context-sensitive nature. The literature reveals variability in how ethics is taught and applied across settings and highlights underexplored areas, including oncology, end-of-life care, digital health and artificial intelligence, and equity, diversity, and inclusion. Findings emphasize the need to strengthen ethics education, reinforce the application of existing codes of ethics, and provide organizational support for ethical deliberation. This synthesis provides a foundation for future research and can inform curricular development, clinical practice, and policy initiatives in physical therapy ethics.

## Introduction

In the medical and health care context, the responsibilities of professionals extend across several dimensions: moral (guided by one’s personal moral principles), ethical (based on principles shared by the professional community), deontological (defined by professional codes and duties, where actions are evaluated by rule-following), and legal (in accordance with civil and criminal law).^1^ This study aims to highlight the ethical dimension of clinical physical therapy practice, as explored by medical ethics and bioethics disciplines.

Medical ethics, through a logical and analytical approach, identifies the principles that guide the clinical practice of health care professionals.^2^ The 4 biomedical principles—beneficence (the good clinician identifies clinically appropriate interventions to protect the patient’s health and life), non-maleficence (the good clinician does not harm the patient’s health or life), autonomy (the good clinician respects the patient’s will to accept or refuse a proposed clinical intervention after evaluating its ethical proportionality), and justice and equity (the good clinician does not discriminate among patients and guarantees fair access to care)—were articulated by Beauchamp and Childress and have since become a foundational framework for clinical decision-making.^3^^,^^4^ Bioethics, as a broader interdisciplinary field, encompasses not only these principles but also the ethical challenges that arise in clinical practice in light of biomedical and biotechnological progress, employing methods of rational argumentation to address them.^5^

In their professional practice, physical therapists may encounter various ethical problems.^6^ These may be shared with other health care professionals or may be specific to physical therapy—particularly those that emerge in a care relationship where physical interaction is a central element.^7^ Physical therapy is characterized by a rehabilitative process that develops over time, requiring a continuous relationship between the professional and the patient.^8^^,^^9^ Within this relationship, physical therapists are called upon to build trust, responsibly manage informed consent, and balance the obligation to provide effective treatment with the need to respect patient preferences.^10^^,^^11^ The element of physical contact, central to physical therapy practice, also raises ethical concerns regarding proxemics and touch.^12^^,^^13^ Physical therapists must be able to modulate physical interaction, ensuring that contact is always appropriate, necessary, and accepted, considering individual and cultural sensitivities.^14^

Prior syntheses of ethics in physical therapy—most notably Swisher’s retrospective analysis of the literature from 1970 to 2000—described how scholarship evolved across decades, characterized dominant ethical approaches, with a strong emphasis on principles-based philosophical work and a later rise of social scientific studies. It identified recurring themes and gaps, such as limited empirical evidence and unaddressed cultural dimensions.^15^ This work provided an essential foundation for the profession. The present scoping review expanded on the literature examined by Swisher, incorporating a broader range of sources (peer-reviewed journals, grey literature, and academic library resources), and extends the historical period considered and the aims.

Hence, the aim of this scoping review was to systematically map ethical and bioethical issues addressed in the physical therapy literature, identify the research methodologies employed, and highlight existing knowledge gaps to inform education, practice, and policy.

## Methods

This systematic scoping review followed the methodological guidance provided by the Joanna Briggs Institute (JBI) and is reported in accordance with the Preferred Reporting Items for Systematic Reviews and Meta-Analyses extension for Scoping Reviews (PRISMA-ScR).^16^^,^^17^ The protocol for this systematic scoping review was published on medRxiv.^18^ This review is not a direct update of prior retrospective analyses (eg, Swisher 1970-2000)^15^; rather, it applies a scoping-review methodology to broaden coverage, triangulate data sources, and introduce a complementary analytical lens focused on mapping ethical and bioethical issues through triangulation of sources.

### Research team

The research team comprised 4 physical therapists and 1 philosopher, all of whom have expertise in qualitative and quantitative research, as well as evidence synthesis. One physical therapist (F.P.) holds a master’s degree in philosophy, while another (G.B.) is pursuing a PhD in neuroscience, with a specific focus on bioethics applied to rehabilitation. One physical therapist (S.B.), PhD, is a research fellow and acted as methodologist to ensure methodological rigor. Another team member (M.T.), PhD, is an associate professor of physical therapy with extensive experience in clinical and academic research. The philosopher (S.P.M.) holds a PhD in bioethics. This interdisciplinary composition ensures a comprehensive and context-sensitive approach to the ethical aspects of physical therapy practice.

### Eligibility criteria

Eligibility for study inclusion was determined using the Population, Concept, and Context (PCC) framework outlined by JBI.^16^

### Population

Studies focusing on physical therapists as professionals and physical therapy as a discipline were included. Research investigating ethical challenges and bioethical issues in physical therapy clinical practice was considered.

Studies examining other health care professionals were excluded unless they explicitly discussed ethical issues concerning physical therapists. Likewise, studies from the patient’s perspective were only included if they directly assessed physical therapists’ ethical concerns.

### Concept

The primary focus of this review was ethics and bioethics within physical therapy practice. Studies had to address ethical problems, principles, or challenges in the field. Research focusing exclusively on technical or procedural aspects of physical therapy, without an ethical dimension, was excluded.

### Context

No restrictions were applied concerning geographical location, demographic, social, or cultural factors. Studies from diverse health care systems and settings were included.

### Types of studies

The disciplinary foundation of medical ethics and bioethics is moral philosophy. Therefore, these fields—though applied to scientific domains such as medicine and health care—remain fundamentally humanistic and theoretical in nature. The main research methods in ethics are descriptive and normative. Depending on the method applied, different types of studies are identified in the literature.

In line with the objectives of this systematic scoping review and the inherently philosophical nature of ethics and bioethics, we included a wide range of study types. Specifically, we considered both descriptive and normative studies, reflecting the 2 main methodological approaches to ethical inquiry.

Descriptive (or empirical) ethics employs what we can call qualitative or social science-based studies—such as interviews, focus groups, observational research, or surveys—to investigate the ethical perceptions of physical therapists, that is, what these professionals perceive or believe to be good or bad (ethical principles) in their daily practice.^19^

Normative ethics proposes a thesis of good or bad (ethical thesis)—which can be entirely independent of the results of descriptive ethics—that the author argues dialectically through a logical-rational analysis. These studies may be presented in the form of reviews, philosophical analyses, or editorials. By examining the literature on ethics and bioethics applied to the field of physical therapy, several studies of this kind were identified, which represent central works of the ethical discourse.

Studies were excluded if they (1) did not explicitly address ethics or bioethics, (2) were not aligned with the scope of physical therapy, (3) were published in non-scholarly formats (eg, commentaries, letters, or duplicates), or (4) were unavailable in full text despite repeated attempts to obtain them. In relation to criterion (2), “not aligned with the scope of physical therapy” referred, for example, to articles addressing ethical issues exclusively in other health professions without relevance to physical therapy, or to legal/administrative discussions without clinical or educational implications for physical therapy.

All included studies—regardless of type—were classified into 1 of the 2 broad categories: descriptive (empirical) or normative ethics, based on their methodological approach.

### Search strategy and information sources

The International Prospective Register of Systematic Reviews (PROSPERO) was consulted to ensure no existing systematic reviews covered this topic. Then, a literature search was conducted employing a data triangulation strategy by combining searches across bibliographic databases, grey literature, and specialized library resources. This approach was intended to enhance comprehensiveness and reduce the risk of missing relevant sources.

The following databases were consulted: PubMed, Embase, Cochrane Central, CINAHL, PsycINFO, and PEDro up to October 2024. These databases were selected based on their comprehensive coverage of health-related research, following established Cochrane recommendations.^20^ The search strategy was developed for PubMed and adapted for each database. The complete list of search terms is provided in Supplementary Material 1 (Research String). No date or language restrictions were applied. The grey literature search followed the guidelines of the Canadian Agency for Drugs and Technologies in Health (CADTH).^21^ Finally, to ensure a comprehensive analysis of bioethics literature related to physical therapy, an extended search was conducted on non-bibliographic sources, including both scientific databases and library resources. To this end, we collaborated with the University of Verona (Verona, Italy) librarians to access the Universe portal, which includes non-bibliographic sources. This allowed us to search for books, book chapters, conference proceedings, and websites related to the topic. If necessary, study authors were contacted for missing data.

### Study selection

All identified records were uploaded to Covidence, where duplicates were automatically removed (Covidence systematic review software, Veritas Health Innovation, Melbourne, Australia. Available at www.covidence.org). Two independent reviewers (G.B., F.P.) screened titles and abstracts in a blinded manner. A calibration exercise was performed on a random 10% of records to ensure interrater reliability. As the agreement was above 90%, no refinement of the inclusion and exclusion criteria was needed, and the second pilot test was not required. Conflicts were resolved through consultation with a third reviewer (S.B.). A PRISMA flow diagram was used to document the selection process.

### Data extraction

Data were extracted using a modified version of the JBI Standardized Data Extraction Form. Consistent with the iterative nature of scoping reviews, the list of extracted variables was refined during the process to include those most consistently reported across studies and most relevant for addressing the review questions:

- Authors
- Year of publication
- Country of origin
- Title
- Ethical inquiry type
- Study design
- Main ethical topics
- Domains of physical therapy practice

This adjustment ensured a more reliable and coherent dataset while maintaining fidelity to the overarching goals of the review. Two independent reviewers (G.B., F.P.) conducted the data extraction.

### Data synthesis

A narrative synthesis was conducted to classify ethical issues in physical therapy. The grouping was based on the predominant topic addressed in each study, as identified through careful reading and analysis of the content.

Articles were grouped into the following key thematic categories:

- Ethical theories: foundational ethical principles and logical-analytical arguments guiding physical therapy.
- Ethical reasoning: how physical therapists identify, interpret, and address ethical issues in clinical practice (including true dilemmas, value conflicts, and experiences of moral distress).
- Ethical reasoning and education: works that address the development of ethical competence through education, training, and reflective learning.
- Ethical perception: what physical therapists perceive to be good or bad in their professional practice.
- Ethics of care relationship: ethical dimensions of the therapeutic relationship, including trust, communication, and boundaries.
- Justice and equity in clinical ethics: fairness, access to care, and systemic inequalities in physical therapy.
- Codes of ethics: content, use, and impact of formal professional codes within the field.

Findings were summarized to highlight how ethical considerations are manifested across different aspects of physical therapy practice. Gaps in the literature were identified, and potential areas for future research were suggested. The results were presented using tables to provide a representation of ethical topics and methodological trends.

In the data synthesis and discussion, we used the term *ethical dilemma* narrowly to indicate situations in which core ethical principles conflict, such that any available option violates an important value. We use *ethical problem* or *ethical issue* as umbrella terms for value-laden situations that may include—but are not limited to—true dilemmas, value conflicts, and experiences of *moral distress* (ie, when clinicians judge the right action but are constrained from acting on it). This policy ensures consistent and accurate usage throughout the manuscript.

#### Deviations from the protocol

While this systematic scoping review was conducted in accordance with the methodological framework established in the published protocol, some deviations occurred during the review process.^18^ First, to ensure a more comprehensive exploration of ethical discourse, we decided to include editorials and narrative reviews, provided they offered substantial normative analysis or critical reflection. This decision was not explicitly outlined in the original protocol but was deemed methodologically coherent given the philosophical nature of the research topic. Finally, although the protocol stated a broad search strategy, the review extended its scope to include non-bibliographic sources by accessing academic library systems through the Universe portal. These deviations were consistent with the iterative nature of scoping reviews and contributed to a more robust mapping of the ethical literature in physical therapy.

## Results

**Figure 1:**
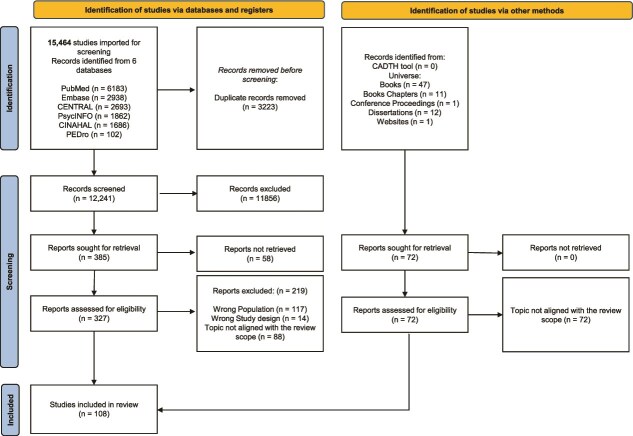
PRISMA Flow Diagram of Study Selection. The diagram illustrates the number of records identified, screened, excluded, and included, along with reasons for exclusion (eg, lack of explicit focus on ethics/bioethics, misalignment with physical therapy, non-scholarly formats, or full-text unavailable). Abbreviation: CADTH = Canadian Agency for Drugs and Technologies in Health.

A total of 15,464 records were identified through database searching. After removing 3223 duplicates, 12,241 records were screened by title and abstract. Of these, 11,856 were excluded as irrelevant, leaving 385 studies for full-text assessment. Among these, 219 records were excluded for the following reasons: a lack of explicit focus on ethics or bioethics, misalignment with the scope of physical therapy, or publication in non-scholarly formats (eg, commentaries, letters, or duplicates). In addition, 58 full texts could not be retrieved despite repeated attempts through institutional access and direct author contact (via email or ResearchGate). The grey literature search, conducted according to the guidelines of the CADTH, did not yield any additional sources for inclusion. Similarly, the search in bibliographic and library systems dedicated to the humanities resulted in 72 records, all of which were deemed irrelevant and thus excluded from the review. Hence, 108 studies met the inclusion criteria and were included in this review (Figure 1; PRISMA flow diagram). The list of full texts excluded from the review, with reasons, is available in the Supplementary Materials 2—excluded studies.

Of the 108 studies, 50 employed a normative approach (25 ethical analysis, 6 case studies, 11 editorials, 7 reviews, and 1 Delphi study), while 58 were descriptive (22 qualitative studies, 13 surveys, 7 cross-sectional studies, 5 longitudinal studies, 4 observational studies, 5 mixed methods, and 2 Delphi studies). The studies covered various physical therapy domains, with the majority addressing general physical therapy (84), followed by professional ethics (8), geriatric care (3), infectious diseases (4), and other specialized areas. The key ethical topics explored included ethical reasoning (33), ethical reasoning and education (19), ethical theories (12), codes of ethics (8), justice and equity in clinical ethics (8), ethics of care relationships (15), and the perception of ethical issues in physical therapy (13).

A summary of the study characteristics is presented in the Table 1, while a detailed list of the included studies is provided in Supplementary Material 3—detailed list of included studies.

**Table 1:** Summary of study characteristics

Study characteristic	Studies (*n*)
**Ethical inquiry type**	Normative (*n* = 50)
	Descriptive (empirical) (*n* = 58)
**Main ethical topics**	Code of ethics (*n* = 8)
	Ethical reasoning (*n* = 33)
	Ethical reasoning and education (*n* = 19)
	Ethical theory (*n* = 12)
	Ethics of the care relationship (*n* = 15)
	Justice and equity in clinical ethics (*n* = 8)
	Perception of ethical issues in physical therapy (*n* = 13)
**Study design**	Ethical analysis (*n* = 25)
	Case study (*n* = 6)
	Cross-sectional (*n* = 7)
	Delphi (*n* = 3)
	Editorial (*n* = 11)
	Longitudinal study (*n* = 5)
	Mixed-methods (*n* = 5)
	Observational studies (*n* = 4)
	Qualitative study (*n* = 22)
	Review (*n* = 7)
	Survey (*n* = 13)
**Domain of physical therapy**	Generic (*n* = 84)
	Geriatric (*n* = 3)
	Infectious diseases (*n* = 4)
	Intensive care (*n* = 1)
	Musculoskeletal (*n* = 1)
	Neurologic (*n* = 2)
	Palliative care (*n* = 1)
	Pediatric (*n* = 2)
	Professional ethics (*n* = 8)
	Sport (*n* = 2)

### Ethical theory

The studies grouped under this category examine the theoretical foundations of ethical reasoning in physical therapy. Several authors critique the limitations of principle-based or deontological ethics when applied to complex clinical situations.^22^ Instead, they propose a contextual ethics in rehabilitation settings.^23^ For instance, some studies suggest that ethical reflection should consider patients' evolving identities and social contexts, particularly in cases of chronic disability.^22^^,^^23^ Other authors incorporate elements of situational ethics to highlight the moral claims implicit in the care relationship.^24^

To operationalize ethical reasoning in clinical practice, several contributions propose applied frameworks.^25^ One notable example is the “active engagement model,” which combines ethical reflection with clinical decision-making by fostering active listening, critical thinking, and dialogical reasoning within multidisciplinary teams.^25^

Further contributions explored the ethical tensions arising from professional roles, such as the “double agent” dilemma, where physical therapists must navigate obligations to patients and employers.^26^ These tensions are analyzed through classical ethical theories—such as utilitarianism (which focuses on maximizing overall benefit), deontology (which emphasizes duties and prohibitions in professional conduct), and ethical egoism (which considers the interests of the individual decision-maker).^26^ Several studies also integrate utilitarian perspectives into rehabilitation goal planning, especially in resource-limited contexts, arguing for a balance between individual-centered care and broader considerations of distributive justice.^27^

Additionally, some authors critically engage with utilitarian bioethics—particularly the views of Peter Singer—arguing for an ethics of rehabilitation that affirms the intrinsic value of persons with disabilities, independent of their perceived productivity or “quality of life”.^28^

Finally, a few theoretical models are proposed to guide ethical analysis in specific contexts, such as private physical therapy practice.^29^ These models emphasize the need for ethical tools that are sensitive to the relational and communicative aspects of care, advocating for a dialogical approach that moves beyond formal consent to promote mutual understanding and trust.^29^

### Ethical reasoning

The studies included in this category focus on how physical therapists engage with ethical challenges in everyday clinical practice, emphasizing reasoning processes. Rather than applying fixed principles, physical therapists often draw upon implicit moral intuitions, practical experience, and case-specific judgments to resolve ethical issues and conflicts among principles.^30^ Ethical reasoning is portrayed as a dynamic, context-dependent process, shaped by clinical environment, resource constraints, and interprofessional relationships.^31^

However, several contributions highlight the importance of integrating ethical reflection into broader clinical decision-making frameworks.^32^ These studies suggest that ethical reasoning in physical therapy is most effective when embedded in relational modes of thinking, allowing for attention to patient goals.^33^

To support such reasoning, several authors propose structured tools and models. The Realm-Individual Process-Situation (RIPS) model, for example, facilitates ethical analysis by distinguishing between individual, organizational, and societal dimensions of a dilemma.^34^ Other studies emphasize the utility of structured ethical consultations to help clinicians identify value conflicts and clarify justifiable actions.^35^

Importantly, ethical reasoning in physical therapy also reflects tensions between ethical principles and external pressures. Studies in sports medicine, occupational health, and private practice reveal that financial incentives, institutional policies, and performance expectations can challenge physical therapists’ ability to act according to their ethical commitments within a sort of “moral compromise.”^36^

Finally, several contributions argue for enhancing ethical reasoning through continuous ethics education, interprofessional dialog, and reflective practice.^37^

### Ethical reasoning and education

This category encompasses studies that investigate the development, effectiveness, and pedagogical strategies of ethics education in physical therapy. A central concern is how educational interventions influence students' ethical reasoning across academic and clinical settings. Several contributions employ longitudinal or pre-post designs using standardized instruments such as the Defining Issues Test (DIT).^38–40^ While some studies demonstrate significant improvement in postconventional ethical reasoning following intensive ethics courses grounded in transformative learning theory, others find little to no measurable change over time, suggesting limitations in conventional curricula.^38–40^

Case-based learning (CBL) emerges as a widely used strategy, promoting ethical reflection through discussion of real-life scenarios.^41^ When introduced early in training, this method fosters critical thinking and enhances moral sensitivity.^41^^,^^42^ Where there is active student participation in identifying and analyzing ethical problems, the results are promising.^42^

Formal instruction in bioethics appears to have the effect of enhancing students’ confidence in addressing ethical issues and improving interprofessional relational competence.^40^

Comparative studies between students and professionals indicate that practicing clinicians tend to employ more mature moral reasoning patterns than students, likely due to accumulated clinical experience and the demands of real-world decision-making.^43^ These findings underscore the importance of mentorship, reflective practice, and situated learning in the workplace as key components of ethics education.^44^^,^^45^

Clinical placements and clinical education experiences are repeatedly identified as contexts in which ethical awareness is tested and refined. Students frequently encounter ethical tensions, and they often feel ill-equipped to resolve them.^44^^,^^46^^,^^47^ Reflective journaling and narrative analysis reveal gaps in the application of ethical principles and highlight the need for better integration of academic instruction with clinical practice.^46^

Finally, cultural and contextual variables play a role in shaping ethical priorities and reasoning. Some studies identify shifts in value hierarchies across academic years—such as an increasing emphasis on equity and professional responsibility—while others note disciplinary differences in ethical preferences, with physical therapist students tending to favor collaborative and relational approaches to ethical challenges.^41^

Collectively, these studies suggest that ethics education in physical therapy requires more than formal instruction: it demands pedagogical approaches that are experiential, reflective, and context sensitive.

### Perception of ethical issues

Studies in this category explore how physical therapists perceive ethical issues in their daily practice.

Qualitative research conducted internationally reveals considerable variability in how ethical problems are perceived, shaped by local health care structures, regulatory frameworks, and sociopolitical environments.^48–50^ In particular, practitioners report feeling constrained by insufficient ethical guidance in relation to ethical codes.^48^^,^^51^

Perceptions of ethical problems also differ across professional settings and domains. In hospital environments, physical therapists commonly identify issues related to justice, resource allocation, and professional autonomy.^49^^,^^52^ These concerns were especially pronounced during the COVID-19 pandemic, and in low-resource countries.^49^^,^^51^

Sociocultural variables play a significant role in shaping ethical sensitivity and principles. Cross-sectional studies identify gender-related differences in empathic engagement and interpersonal sensitivity, with female physical therapists often demonstrating higher ethical awareness in relational domains.^53^ In certain contexts, private sector professionals report greater alignment with patient autonomy, whereas public sector practitioners prioritize collective needs.^54^ Additionally, differences can be observed across national boundaries and educational backgrounds.^54^

Ethical problems are not limited to patient care but extend to interprofessional relations and organizational dynamics. Physical therapists frequently perceive tensions arising from medical hierarchies, unclear role definitions, and power imbalances, particularly when clinical judgment is overridden by institutional directives.^55^

Several studies emphasize the perceived gap between academic ethics education and real-world ethical challenges. While physical therapist educators recognize the importance of ethics and advocate for greater curricular integration, practicing clinicians often report unmet needs in ethical training.^50^

### Ethics of the care relationship

This category explores the ethical dimensions embedded in the care relationship between physical therapists and patients. Studies emphasized that ethical physical therapy practice extends beyond technical competence to include relational sensitivity and a commitment to fostering patient self-determination.

A central theme across the literature is the ethical complexity of touch, corporeality, and intimacy, particularly in the treatment of elderly or vulnerable patients. These interactions require physical therapists to manage professional boundaries while cultivating a sense of safety and respect, often in the absence of explicit ethical training.^7^^,^^13^

Another key focus concerns the models of the therapeutic relationship, contrasting hierarchical or paternalistic models with a patient-centered care relationship.^56^

The concept of informed consent emerges as a pivotal ethical practice. Some physical therapists view it primarily as a formal requirement or a means to ensure legal safeguard or therapeutic adherence, while others advocate for a dialogic process that supports patient agency and autonomy.^57^

Several studies also examine moral distress experienced by physical therapists when constraints such as limited time, inadequate staffing, or institutional protocols, hinder their ability to provide ethical care aligned with their professional principles.^58^

The asymmetry of knowledge and power in the physical therapist-patient relationship is identified as a persistent challenge. To address this, the literature calls for greater attention to communication and patient empowerment.^57^^,^^59^^,^^60^

### Justice and equity in clinical ethics

This category focuses on ethical issues related to justice, fairness, and equity in physical therapy practice, particularly in relation to access to care and resource allocation.

Several studies explore the tensions between clinical responsibilities and systemic constraints—such as institutional productivity targets, staffing shortages—that may prioritize efficiency or financial metrics over quality and equity of care.^61^^,^^62^

Ethical problems surrounding resource allocation are particularly pronounced in public or resource-limited settings, where physical therapists must make difficult decisions about treatment prioritization and duration.^62^

From a theoretical perspective, scholars have proposed moving beyond traditional distributive models of justice toward approaches that consider the real opportunities individuals must achieve health and well-being.^63^

Relatedly, some studies advocate for a needs-based approach to justice in health care, particularly in systems like Sweden’s, where universal coverage supports more consistent ethical reasoning around equitable care.^64^

The literature also highlights the concept of social responsibility in physical therapy, urging professionals to engage with communities and contribute to health equity beyond the clinical setting.^63^ This includes addressing structural barriers such as provider shortages, limited access to rehabilitation in rural or underserved areas, and sociocultural stigmas.

Educational interventions are seen as essential to preparing future professionals for justice-oriented practice. Experiential learning models—such as critical reflection—are proposed to foster ethical awareness and empower students to address inequities within and beyond clinical environments.^65^

### Codes of ethics

This category explores the development, application, and interpretation of codes of ethics in physical therapy, examining their role in guiding professional behavior, shaping identity, and addressing ethical challenges. Codes of ethics—distinct from clinical guidelines or legal regulations—articulate ethical duties and prohibitions in professional behavior within clinical practice.

Research assessing physical therapists’ awareness and application of ethical codes revealed generally good levels of familiarity, particularly among older and more experienced professionals.^66^

Other studies pointed to a gap between formal codes and everyday practice, with physical therapists often relying on intuitive reasoning or personal principles in ethically complex situations.^67^^,^^68^ These findings highlight the limited use of codified ethical principles in daily decision-making, prompting calls for structured training programs.^67^^,^^69^

Several contributions examine how ethical codes are developed and revised, often through participatory methods.^68^ Some studies emphasized that codes of ethics should be actively internalized during professional formation.^69^ Indeed, rather than serving as static rulebooks, ethical codes are dynamic tools that evolve with changing societal expectations and professional contexts, demanding ongoing reflection, critique, and education to remain relevant and impactful.^70^

From a historical perspective, the early evolution of ethical codes in physical therapy reveals their strategic use in legitimizing the profession. One study traced how the first code of the American Physical Therapy Association (APTA) (1918-1935) prioritized alignment with the male-dominated medical profession and omitted reference to patient-centered principles, reflecting the profession’s struggles with gendered power dynamics and institutional recognition.^71^

## Discussion

The findings of this systematic scoping review underscore the complexity of ethical and bioethical issues in physical therapy. This review identifies the types and foci of studies, maps the principles, theories, and perspectives most frequently discussed, and synthesizes recurring topics and gaps across decades of scholarship. Drawing on prior work, our analysis corroborates patterns noted by Swisher—such as the earlier dominance of principles-based approaches and the later rise of social scientific studies.^15^ By triangulating diverse sources, this review extends earlier retrospective analyses and provides a comprehensive, descriptive overview of the field’s evolving ethical discourse.

Ethical problems permeate various aspects of clinical practice, including professional conduct, patient care, justice and equity, and decision-making frameworks.^44^^,^^72^ The review confirms that ethical reasoning plays a pivotal role in guiding physical therapists through these challenges, yet also highlights significant gaps in training, inconsistencies in ethical application, and structural barriers that complicate clinical judgment. ^45^ These inconsistencies in ethical application include variations in how physical therapists interpret and prioritize core ethical principles, often influenced by contextual factors like workplace norms, cultural expectations, and differing levels of ethical preparedness. Furthermore, structural barriers complicating clinical judgment encompass organizational constraints such as time pressure, productivity demands, lack of interdisciplinary collaboration, and institutional policies, thereby limiting the physical therapists’ ability to make ethically sound decisions.

A key issue emerging from the review concerns the development and integration of ethical reasoning in physical therapy.^39^ While physical therapists frequently encounter ethically ambiguous situations, their ability to manage them effectively is closely tied to their formal ethics education and exposure to structured frameworks.^42^ Without these supports, clinicians often rely on intuitive or ad hoc approaches that lack consistency. The literature supports the adoption of pedagogical strategies such as CBL and interdisciplinary training, yet highlights the limited standardization of ethics education within physical therapist curricula.^41^ Addressing this gap could strengthen clinicians' confidence, promote reflective practice, and enhance their capacity to navigate ethically challenging situations with consistency and integrity, thereby fostering a workforce that is better equipped to sustain ethical decision-making under pressure.^32^

Another relevant dimension relates to the function and status of codes of ethics in physical therapy. While these codes offer a foundational reference for professional conduct, their application in daily practice differs significantly between national contexts—such as differences in legal enforceability across countries—and institutional contexts, which refer to the specific organizational settings where physical therapists work (eg, hospitals, private practices, academic institutions).^68^^,^^70^ In countries like Canada and Australia, codes of ethics are embedded within regulatory frameworks and hold binding authority, whereas in other contexts, such as parts of Europe and Asia, they function more as aspirational or advisory documents without formal enforcement mechanisms.^69^ There are also cases like Italy, where the Code of Ethics for physical therapists is not a state law; however, it is binding for professionals under penalty of disciplinary sanctions imposed by the professional association. Ongoing professional development—meaning continuous learning opportunities that include ethics training and reflection on real-world dilemmas—plays a crucial role in helping clinicians understand, apply, and internalize these codes. ^70^ In the United States, the APTA Code of Ethics similarly serves as a central professional reference. Although not enacted as federal law, it is widely integrated into state-level regulatory frameworks—often through alignment with the Federation of State Boards of Physical Therapy’s Model Practice Act—and thus functions as a binding standard within many jurisdictions. This alignment reflects the Model Practice Act’s recommendation that state statutes incorporate ethical standards directly into regulatory language, a process already adopted or underway across multiple states in the United States.^70^

Justice and equity emerged as particularly salient ethical concerns, especially in contexts of limited resources, systemic inequities, and institutional constraints.^73^ Many physical therapists report ethical distress when unable to deliver equitable care due to bureaucratic or economic pressures.^61^^,^^62^ Addressing these challenges requires expanding the ethical lens beyond individual patient care to include social determinants of health, health policy, and institutional priorities.^74^ Mechanisms such as clinical ethics committees, reflective practice groups, and interprofessional collaboration could support physical therapists in navigating these complex dynamics.^63^

The ethics of the care relationship also warrant close attention.^7^ The embodied and interpersonal nature of physical therapy raises unique ethical challenges, particularly in relation to physical touch and the maintenance of professional boundaries—that is, the ability to establish respectful and appropriate therapeutic relationships without overstepping into the patient's personal or emotional space.^75^ These dynamics are especially relevant in situations involving vulnerable populations or intimate treatment settings. Moreover, physical therapists, like other health care professionals, have an ethical duty to inform and communicate with patients to ensure the exercise of their self-determination.^60^^,^^76^ Achieving this requires ethical training that prioritizes relational competence, sensitivity to context, and a dialogical approach to care.

Sociocultural factors significantly shape how ethical issues are perceived and managed in physical therapy.^48^ For instance, concepts such as patient autonomy, confidentiality, or professional boundaries may be interpreted differently across cultural settings. International studies reveal notable differences in ethical priorities (such as a stronger emphasis on individual rights in Western countries versus collective responsibility in certain Asian contexts) and interprofessional relationships (in hierarchical health care systems—such as those in Italy, India, or Japan—physical therapists may experience constraints on their clinical judgment, for example when decisions are predominantly physician-driven or when institutional protocols override their ethical concerns; by contrast, more collaborative or decentralized systems, such as those in the Netherlands or Scandinavia, may allow for greater professional autonomy).^54^ Several ethical domains appear underexplored in the literature and warrant further attention. For example, ethical issues in oncological and end-of-life care remain insufficiently investigated, despite their high ethical salience for physical therapists.^77^ These settings often involve complex decisions around quality of life, non-maleficence, informed consent, and therapeutic goals that may shift from curative to palliative.^76^

The integration of emerging technologies in physical therapy also introduces novel ethical concerns.^78^ Developments in artificial intelligence, tele-rehabilitation, and assistive technologies raise questions about privacy, data protection, and the evolving nature of the therapist-patient relationship. These technological shifts necessitate careful ethical scrutiny to ensure that innovation supports, rather than undermines, therapeutic integrity and patient engagement.

Equity, diversity, and inclusion (EDI) in physical therapy practice remain insufficiently studied from an ethical perspective. There is a need to explore how linguistic, cultural, and socioeconomic barriers affect access to rehabilitation services, particularly for underserved populations.^57^ Additionally, ethical concerns related to care for lesbian, gay, bisexual, transgender, queer, and other diverse identities (LGBTQIA+), including respect for gender identity and sexual orientation, remain underrepresented.^51^^,^^74^^,^^77^ Recent scholarship has begun to address these gaps by calling for affirming and inclusive practice guidelines for people who identify as LGBTQIA+, strengthening cultural competence in clinical encounters, and advocating for curricular reforms grounded in justice, EDI principles.^79–81^ Structured education and training on cultural competence, inclusivity, and anti-discrimination should be prioritized to foster equitable and respectful care. In conceptual terms, it is important to note that within this review, justice is treated as a broader bioethical principle that cuts across multiple domains, whereas EDI reflects more specific, practice-oriented expressions of justice in contemporary clinical, educational, and organizational contexts. Recognizing this relationship helps clarify why the 2 areas appear in different sections of the analysis while remaining conceptually interconnected.

Altogether, this review carries significant practical implications. The synthesis of ethical and bioethical issues underscores the need to enhance the integration of ethical reasoning in entry-level physical therapist curricula, ensuring that students are equipped to address complex ethical dilemmas in clinical practice. This effort can entail the inclusion of structured ethics modules, the use of CBL, and the adoption of assessment tools that explicitly evaluate ethical reasoning skills.^82^ At the clinical and organizational level, the findings underscore the importance of providing structural supports—such as ethics consultation services, reflective practice groups, and interprofessional dialog—that enable physical therapists to manage ethically challenging situations without isolation. Such mechanisms can help translate ethical reflection into consistent practice and support a culture of ethical accountability within health care institutions.^83–85^ In addition to these practical considerations, the review highlights how the existing literature on ethics in physical therapy reflects recurring themes—such as the therapeutic relationship, the embodied nature of care, and the profession’s intermediary role between patients and health systems—that are particularly relevant in rehabilitation contexts. These contributions can be interpreted as contextual applications of medical and bioethical principles, adapted to the specific features of physical therapy practice.

At the same time, this synthesis reveals a lack of awareness in the literature regarding several ethically relevant areas. Ethical issues in oncological and end-of-life care, in digital health and artificial intelligence, and in the domains of EDI remain underexplored in the literature despite their growing salience for physical therapy practice. Emerging research on LGBTQIA+ health, structural determinants of inequity, and antiracism in physical therapy underscores the urgency of addressing these gaps through more systematic empirical and educational scholarship.^86^^,^^87^ Making sense of this body of work, therefore, requires not only cataloging existing contributions but also spotting these gaps in the literature to inform future studies. Taken together, the most substantial gaps concern: (1) the limited ethical analysis of inequities affecting underserved communities—including LGBTQIA+ individuals and communities facing linguistic, cultural, or socioeconomic barriers; (2) the scarcity of empirical studies addressing justice-related issues in rehabilitation access and outcomes; and (3) the underdeveloped ethical scholarship on emerging areas such as oncological and end-of-life care, digital health, and artificial intelligence.

In addition, several priorities for future scholarship can be identified. First, there is a need to establish clear and shared definitions of ethical concepts as they apply specifically to physical therapy—for example, what constitutes an “ethical dilemma” in hands-on therapeutic encounters, how “moral distress” is experienced when organizational constraints prevent equitable care, or how the notion of a “care relationship” should be framed in a profession where bodily contact and continuity of interaction are central. Establishing such definitions would enable consistent communication across studies, improve the design of ethics education within physical therapist curricula, and strengthen the comparability of research findings. Second, descriptive research—that is, studies investigating how physical therapists experience, interpret, and address ethical challenges in practice—should be expanded to include diverse cultural and geographical settings, particularly non-Western health care systems, in order to capture the global nature of ethical challenges in physical therapy. Ultimately, future research should address emerging issues such as the use of artificial intelligence and concerns related to EDI. Progress in these areas could provide a more comprehensive and globally relevant understanding of ethical and bioethical issues in physical therapy. Attention to features specific to physical therapy—such as the embodied and hands-on nature of care, the frequency and continuity of encounters, the dual role between prescriptions and patients’ goals, and the educational dimension of adherence and empowerment—can help future descriptive research better capture how ethical challenges are experienced and managed in practice.

### Limitations and future directions

While this systematic scoping review provides a comprehensive overview of ethics in physical therapy, certain limitations must be acknowledged. Including both descriptive (empirical) and normative bioethics studies introduces heterogeneity in the analyzed literature, which may impact comparability. Another limitation is the potential underrepresentation of ethical issues specific to non-Western health care contexts. While this review aimed to include diverse perspectives, the predominance of studies from high-income countries is evident, with approximately 75% of the data coming from North America and Europe. This geographic imbalance is a well-documented structural limitation across many professional fields; however, it holds particular significance in ethics, where cultural, social, and institutional norms deeply influence how ethical issues are defined, prioritized, and addressed. In addition, our search strategy followed the predefined string reported in the published protocol. As a consequence, broader constructs such as professionalism, professional formation, or professional development were not included as search terms. While this choice ensured methodological consistency with the protocol, it may have limited the retrieval of studies primarily indexed under those categories. Future reviews should examine these constructs in dedicated analyses, allowing focused exploration of their ethical dimensions. Finally, although we systematically searched grey literature sources using a structured checklist, it is not feasible to capture all possible grey literature materials. Widely used educational textbooks in physical therapy may not have been included, despite their acknowledged influence on ethics education within the profession. These include key texts such as Patient–Practitioner Interaction,^88^ Professional Issues and Ethics in Physical Therapy,^89^ and Ethical Dimensions in the Health Professions.^90^

## Conclusion

This systematic scoping review presents a descriptive synthesis of ethical and bioethical issues in physical therapy across various domains, including ethical reasoning, justice and equity, codes of ethics, the care relationship, and the perception of ethical problems in practice. These themes reflect how the 4 foundational biomedical principles—autonomy, beneficence, non-maleficence, and justice—are interpreted and operationalized within contemporary rehabilitation contexts. Although ethical challenges are pervasive, targeted strategies—such as structured ethics education and organizational support mechanisms (eg, ethics consultation services, reflective practice groups, and policies that enable deliberation)—can strengthen clinical decision-making and promote patient-centered, equitable care. The review identifies several underexplored areas—including oncology and end-of-life rehabilitation, digital health and artificial intelligence, and EDI—as well as cross-cultural gaps that warrant further descriptive research. These gaps carry distinct implications for education, practice, and policy. In education, they highlight the need to reinforce ethics teaching, cultural competence, and structured opportunities for ethical reflection within physical therapist curricula. In clinical practice, they underscore the value of accessible ethical resources and institutional supports to assist clinicians in navigating complex dilemmas. At the policy level, the findings point to opportunities to refine ethical standards, strengthen regulatory guidance, and promote more equitable access to rehabilitation services.

Overall, these contributions are intended to inform curricula, professional development and research, and service organizations.

## Supplementary Material

PTJ-2025-0428_R2_Supplementary_Materials_pzag011

## Data Availability

The datasets generated during and/or analyzed during the current study are available from the corresponding author on reasonable request.
