# Supplementary material for: Ethical and bioethical issues in physical therapy: A systematic scoping review
Source: Phys Ther. 2026 Feb 2;106(3):pzag011. doi: 10.1093/ptj/pzag011 (PMC13017120; doi:10.1093/ptj/pzag011)
Supplement: PTJ-2025-0428_R2_Supplementary_Materials_pzag011 [file ptj-2025-0428_r2_supplementary_materials_pzag011.pdf]

Supplementary Material 1 – Research String

Medline via Pubmed consulted in date 17/10/2024				
Population	1	**Physical Therapy Modalities**	**[Mesh Term]**	187,529
	2	**Physical Therapy Specialty**	**[Mesh Term]**	3,087
	3	**Rehabilitation**	**[Mesh Term]**	369,104
	4	Rehabilitation	[Title/Abstract]	842,130
	5	Physiotherap*	[Title/Abstract]	83,274
	6	"Physical therap*"	[Title/Abstract]	123,518
	7	"Physical rehabilitat*"	[Title/Abstract]	3,744
	8	"Rehabilitation therap*"	[Title/Abstract]	4,778
	9	Physiotherapist	[Title/Abstract]	5,056
	10	Physiotherapists	[Title/Abstract]	8,527
	11	“Physical therapist”	[Title/Abstract]	3,808
	12	“Physical therapists”	[Title/Abstract]	6,165
	13	1 OR 2 OR 3 OR 4 OR 5 OR 6 OR 7 OR 8 OR 9 OR 10 OR 11 OR 12		571,211
Concept	14	**Ethics**	**[Mesh Term]**	158,840
	15	**Ethics Consultation**	**[Mesh Term]**	1,435
	16	**Ethics, Research**	**[Mesh Term]**	9,169
	17	**Codes of Ethics**	**[Mesh Term]**	5,564
	18	**Principle-Based Ethics**	**[Mesh Term]**	34,111
	19	**Ethics, Clinical**	**[Mesh Term]**	65,200
	20	**Ethics, Professional**	**[Mesh Term]**	75,373
	21	**Ethics, Nursing**	**[Mesh Term]**	10,626
	22	**Ethics, Medical**	**[Mesh Term]**	48,722
	23	**Ethical Theory**	**[Mesh Term]**	3,678
	24	**Bioethics**	**[Mesh Term]**	12,174
	25	Ethics	[Title/Abstract]	99,889
	26	Bioethics	[Title/Abstract]	17,089
	27	"Professional ethics"	[Title/Abstract]	9,808
	28	“Ethical issue*”	[Title/Abstract]	15,479
	29	"Ethical dilemma*”	[Title/Abstract]	5,565
	30	"Ethical principle*"	[Title/Abstract]	4,657
	31	"Moral obligation*"	[Title/Abstract]	1,053
	32	"Moral issue*"	[Title/Abstract]	789
	33	“Moral principle*”	[Title/Abstract]	541
	34	"Moral dilemma*”	[Title/Abstract]	1,333
	35	“Ethical decision-making”	[Title/Abstract]	1,705
	36	“Moral decision-making”	[Title/Abstract]	492
	37	"Ethical consideration*"	[Title/Abstract]	8,319
	38	“Ethical challenge*”	[Title/Abstract]	4,026
	39	“Moral consideration*”	[Title/Abstract]	281
	40	“Moral challenge*”	[Title/Abstract]	203
	41	"Bioethical issue*”	[Title/Abstract]	510
	42	“Bioethical dilemma*”	[Title/Abstract]	104
	43	14 OR 15 OR 16 OR 17 OR 18 OR 19 OR 20 OR 21 OR 22 OR 23 OR 24 OR 25 OR 26 OR 27 OR 28 OR 29 OR 30 OR 31 OR 32 OR 33 OR 34 OR 35 OR 36 OR 37 OR 38 OR 39 OR 40 OR 41 OR 42		239,332

	44	13 AND 43	**5,907**

PsycINFO consulted in date 05/11/2024

Physical Therapy Modalities *OR* **MeSH**: Physical Therapy Specialty *OR* **MeSH**: Rehabilitation *OR* **Title**: Rehabilitation *OR* **Title**: Physiotherap**OR* **Title**: "Physical therap*" *OR* **Title**: "Physical rehabilitat*" *OR* **Title**: "Rehabilitation therap*" *OR* **Title**: Physiotherapist *OR* **Title**: Physiotherapists *OR***Title**: "Physical therapist" *OR* **Title**: "Physical therapists" *AND* **MeSH**: Ethics *OR***MeSH**: Ethics Consultation *OR* **MeSH**: Ethics, Research *OR* **MeSH**: Codes of Ethics*OR* **MeSH**: Principle-Based Ethics *OR* **MeSH**: Ethics, Clinical *OR* **MeSH**: Ethics, Professional *OR* **MeSH**: Ethics, Nursing *OR* **MeSH**: Ethics, Medical *OR* **MeSH**: Ethical Theory *OR* **MeSH**: Bioethics *OR* **Title**: "Professional ethics" *OR* **Title**: "Ethical issue*" *OR* **Title**: "Ethical dilemma*" *OR* **Title**: "Ethical principle*" *OR* **Title**: "Moral obligation*" *OR* **Title**: "Moral issue*" *OR* **Title**: "Moral principle*" *OR* **Title**: "Moral dilemma*" *OR* **Title**: "Ethical decision-making" *OR* **Title**: "Moral decision-making" *OR* **Title**: "Ethical consideration*" *OR* **Title**: "Ethical challenge*" *OR* **Title**: "Moral consideration*" *OR* **Title**: "Moral challenge*" *OR* **Title**: "Bioethical issue*" *OR***Title**: "Bioethical dilemma*"

Embase consulted in date 05/11/2024

('physical therapy modalities':ti,ab OR 'physical therapy specialty':ti,ab OR 'rehabilitation':ti,ab OR rehabilitation:ti,ab OR physiotherap*:ti,ab OR 'physical therap*':ti,ab OR 'physical rehabilitat*':ti,ab OR 'rehabilitation therap*':ti,ab OR physiotherapist:ti,ab OR physiotherapists:ti,ab OR 'physical therapist':ti,ab OR 'physical therapists':ti,ab) AND ('ethics':ti,ab OR 'ethics consultation':ti,ab OR 'ethics, research':ti,ab OR 'codes of ethics':ti,ab OR 'principle-based ethics':ti,ab OR 'ethics, clinical':ti,ab OR 'ethics, professional':ti,ab OR 'ethics, nursing':ti,ab OR 'ethics, medical':ti,ab OR 'ethical theory':ti,ab OR 'bioethics':ti,ab OR ethics:ti,ab OR bioethics:ti,ab OR 'professional ethics':ti,ab OR 'ethical issue*':ti,ab OR 'ethical dilemma*':ti,ab OR 'ethical principle*':ti,ab OR 'moral obligation*':ti,ab OR 'moral issue*':ti,ab OR 'moral principle*':ti,ab OR 'moral dilemma*':ti,ab OR 'ethical decision-making':ti,ab OR 'moral decision-making':ti,ab OR 'ethical consideration*':ti,ab OR 'ethical challenge*':ti,ab OR 'moral consideration*':ti,ab OR 'moral challenge*':ti,ab OR 'bioethical issue*':ti,ab OR 'bioethical dilemma*':ti,ab) AND [embase]/lim

Cinhal consulted in date 05/11/2024

((((((((((((MH "Physical Therapy Modalities+")) OR ((MH "Physical Therapy Specialty+"))) OR ((MH Rehabilitation+))) OR (Rehabilitation)) OR (Physiotherap*)) OR ("Physical therap*")) OR ("Physical rehabilitat*")) OR ("Rehabilitation therap*")) OR (Physiotherapist)) OR (Physiotherapists)) OR ("Physical therapist")) OR ("Physical therapists")) AND (((((((((((((((((((((((((((((MH Ethics+)) OR ((MH "ethics consultation+"))) OR ((MH "ethics, research+"))) OR ((MH "codes of ethics+"))) OR ((MH "Principle-Based Ethics+"))) OR ((MH "ethics, clinical+"))) OR ((MH "Ethics, Professional+"))) OR ((MH "ethics, nursing+"))) OR ((MH "ethics, medical+"))) OR ((MH "ethical theory+"))) OR ((MH bioethics+))) OR (Ethics)) OR (Bioethics)) OR ("Professional ethics")) OR ("Ethical issue*")) OR ("Ethical dilemma*")) OR ("Ethical principle*")) OR ("Moral obligation*")) OR ("Moral issue*")) OR ("Moral principle*")) OR ("Moral dilemma*")) OR ("Ethical decision-making")) OR ("Moral decision-making")) OR ("Ethical consideration*")) OR ("Ethical challenge*")) OR ("Moral consideration*")) OR ("Moral challenge*")) OR ("Bioethical issue*")) OR ("Bioethical dilemma*"))

Filters:
Research Article
Peer Reviewd
Exclude Medline Records

PEDro consulted in date 05/11/2024

Abstract and title: Ethics

Supplementary Material 2 – Records excluded with reasons

Author	Year	Title	Reason for exclusion
M Aguillar-Rodriguez	2019	A blended-learning programme regarding professional ethics in physiotherapy students	Topic not aligned with the review scope
JV Stillo	1995	A case in rehabilitation ethics.	No Full Text
JB Lattanzi	2011	A Conceptual Framework for International Service-Learning Course Planning	Topic not aligned with the review scope
AR Egbert	2017	A Framework for Ethical Decision Making in the Rehabilitation of Patients with Anosognosia.	Wrong Population
No authors listed	1995	A guide to physical therapist practice, Volume I: A description of patient management. American Physical Therapy Association.	No Full Text
C Delany	2012	A process of informed consent for student learning through peer physical examination in pelvic floor physiotherapy practice	Topic not aligned with the review scope
M Veras	2024	A rapid review protocol of physiotherapy and occupational therapy telerehabilitation to inform ethical and equity concerns.	Wrong study design
LL Swisher	2002	A retrospective analysis of ethics knowledge in physical therapy (1970-2000)	Wrong study design
T Hartley	2016	A Survey of Current and Projected Ethical Dilemmas of Rehabilitation Counselors.	Wrong Population
E Lind Irgens	2016	Acquired brain injury rehabilitation: dilemmas in neurological physiotherapy across healthcare settings.	Topic not aligned with the review scope
M Laliberté	2015	An analysis of ethics teaching in Canadian physiotherapy and occupational therapy programs	No Full Text
M Laliberté	2015	An in-depth analysis of ethics teaching in Canadian physiotherapy and occupational therapy programs.	Topic not aligned with the review scope
MT Samo	1993	Aphasia rehabilitation: psychosocial and ethical considerations.	Wrong Population
LP Brewster	2006	Application of rehabilitation ethics to a selected burn patient population's perspective.	Wrong Population
J Najem	2018	Assessing Rehabilitation Eligibility of Older Patients: An Ethical Analysis of the Impact of Bias.	Wrong Population
E Marques-Sulé	2021	Attitudes towards learning professional ethics in undergraduate physiotherapy students: A STROBE compliant cross-sectional study.	Topic not aligned with the review scope
LR Cherney	2020	Autonomy and the Patient with Right Hemisphere Cognitive-Communication Deficits: Ethical Considerations in Rehabilitation Practice.	Wrong Population
AFV Badarò	2008	Bioethics and research in physical therapy: approximation and bonds	Wrong study design
AG Scheneiders	2015	Blood, sweat and tears: reclaiming the ethical high ground in sports physiotherapy.	Wrong study design
A Mansbach	2011	Blowing the whistle to protect a patient: a comparison between physiotherapy students and physiotherapists	Topic not aligned with the review scope
S Blanton	2020	Can Reading Tolstoy Make Us Better Physical Therapists? The Role of the Health Humanities in Physical Therapy.	Topic not aligned with the review scope
Z Davidow	2010	Can You Learn Ethics by Watching TV? The Use of TV Shows in Teaching Ethics in Physical Therapy.	No Full Text
M Butler	2008	Care ethics and brain injury.	Wrong Population

G Gard	2003	Changes in life-views and ethical viewpoints during physiotherapy education	No Full Text
Boland Patterson J	2000	Choice: ethical and legal rehabilitation challenges.	Wrong Population
JA Sliwa	2002	Clinical ethics in rehabilitation medicine: core objectives and algorithm for resident education.	Wrong Population
EJ Phipps	1998	Communication and ethics: cardiopulmonary resuscitation in head trauma rehabilitation.	Wrong Population
J Garcia	2009	Comparing two training strategies to increase competence in solving ethical dilemmas.	No Full Text
S Beveridge	2015	Comparison of Ethical Dilemmas Across Public and Private Sectors in Rehabilitation Counseling Practice.	Wrong Population
S Cross	2000	Confidentiality within physiotherapy: perceptions and attitudes of clinical practitioners.	Topic not aligned with the review scope
GR Scofield	1993	Considerations on the ethics of rehabilitation medicine	Wrong Population
A Hudon	2015	Content analysis of the Canadian rehabilitation ethics teaching workshop (crew day)	No Full Text
C Finley	1991	Curriculum survey: ethical and legal instruction -- a report from the APTA Department of Education and the APTA Judicial Committee.	No Full Text
JD Banja	1994	Deception in advertising and marketing: ethical applications in rehabilitation.	Wrong Population
I Novak	2021	Decisionmaking in rehabilitation.	Wrong Population
V V Orzheshkovskii	1987	Deontological problems in a system for the postgraduate training of physiotherapists].	No Full Text
M Clemence	2001	Developing the ethics of placebos in physiotherapy	Wrong study design
P Lee	2015	Development of a decision tool to ethically assign care to the OTA and PTA	Topic not aligned with the review scope
C Carpenter	2002	Dilemmas of practice as experienced by physical therapists in rehabilitation settings...including commentary by Barreca S	No Full Text
SE Roush	2011	Disability reconsidered: the paradox of physical therapy.	Wrong study design
DA Nicholls	2012	Discipline, desire, and transgression in physiotherapy practice	Topic not aligned with the review scope
MJ Young	2024	Disorders of Consciousness Rehabilitation: Ethical Dimensions and Epistemic Dilemmas.	Wrong Population
M Lalibertè	2013	Do conflicts of interest create a new professional norm? Physical therapists and workers' compensation.	Wrong study design
M Aguillar-Rodriguez	2021	Effect of a Programme Based on Professional Ethics in Physiotherapy Students	No Full Text
G Gartland	1987	Essentials of ethics in clinical practice: a communications perspective.	No Full Text
AL Caplan	1987	Ethical & policy issues in rehabilitation medicine.	Wrong Population
KL Kirschner	2012	Ethical challenges of caring for VIPs in the rehabilitation setting: Part II.	Wrong Population
KL Kirschner	2012	Ethical challenges of caring for VIPs in the rehabilitation setting.	Wrong Population
K Naamanka	2023	Ethical competence - exploring situations in physiotherapy practice.	No Full Text

JL Saunders	2007	Ethical complaints and violations in rehabilitation counseling: an analysis of Commission on Rehabilitation Counselor Certification data.	Wrong Population
JE Miller	2015	Ethical concerns identified by physical medicine and rehabilitation residents.	Wrong Population
JM Young	2001	ETHICAL CONCERNS OF STAFF IN A REHABILITATION CENTER	Topic not aligned with the review scope
JF Malec	1996	Ethical conflict resolution based on an ethics of relationships for brain injury rehabilitation.	Wrong Population
R Flanagan	1986	Ethical considerations for the peace activist psychotherapist.	Wrong Population
JK Plummer	1995	Ethical considerations in brain injury rehabilitation: applications to mild traumatic brain injury.	Wrong Population
GR Scofield	1993	Ethical considerations in rehabilitation medicine	Wrong Population
PD Rumrill	1999	Ethical considerations in reporting and publishing rehabilitation research	Wrong Population
JR Bach	1994	Ethical considerations in the management of individuals with severe neuromuscular disorders	Wrong Population
J Haas	1993	Ethical considerations of goal setting for patient care in rehabilitation medicine	Wrong Population
C Christiansen	2001	Ethical considerations related to evidence-based practice.	Wrong Population
SL Hanson	2007	Ethical decision making in rehabilitation: Consideration of Latino cultural factors.	Wrong Population
NR Kirsch	2009	Ethical decision making: Application of a problem-solving model	No Full Text
BP Horowitz	2003	Ethical decision-making challenges in clinical practice.	Wrong Population
BT Vaughn	1998	Ethical dilemmas encountered by private sector rehabilitation practitioners.	Topic not aligned with the review scope
BR Hasselkus	1991	Ethical dilemmas in family caregiving for the elderly: implications for occupational therapy.	Wrong Population
VM Tarvydas	2010	Ethical dilemmas of rehabilitation counselors: results of an international qualitative study.	Wrong Population
JC Hill	2023	Ethical Dilemmas: Current and Projected Concerns Reported by Certified Rehabilitation Counselors.	Wrong Population
R Basevi	2014	Ethical guidelines and the use of social media and text messaging in health care: a review of literature.	Topic not aligned with the review scope
KT Lucke	1998	Ethical implications of caring in rehabilitation.	Wrong Population
F Tasseau	2011	Ethical issues concerning the Leonetti law and its application for people with severe brain damage	Wrong Population
DJ Matthews	1990	Ethical issues encountered in pediatric rehabilitation.	Wrong Population
J Stein	2012	Ethical issues in inpatient rehabilitation length of stay determination.	Wrong Population
PJ Flett	2003	Ethical issues in paediatric rehabilitation.	Wrong Population
J Donders	2013	Ethical issues in pediatric traumatic brain injury rehabilitation.	Wrong Population
MG Kuzczewski	2005	Ethical issues in physical medicine and rehabilitation: Treatment decision making with adult patients	Wrong Population
JF Haas	1994	Ethical issues in physical medicine and rehabilitation. Conclusion to a series.	Wrong Population

RW Richardson	2015	Ethical issues in physical therapy	Wrong study design
HM Robillard	1989	Ethical issues in primary health care: a survey of practitioners' perceptions.	Wrong Population
M Martone	2004	Ethical issues in rehabilitation in the home-care setting.	No Full Text
J Blackmer	2000	Ethical issues in rehabilitation medicine.	Wrong Population
J Banja	2001	Ethical issues in rehabilitation science and medicine	Wrong Population
M Kuczewski	2001	Ethical issues in rehabilitation: conceptualizing the next generation of challenges.	Wrong Population
RB Purtilo	1988	Ethical issues in teamwork: the context of rehabilitation.	No Full Text
J Sim	1997	Ethical issues in the management of persistent vegetative state.	Wrong Population
J Matthes	2013	Ethical issues in using deception to facilitate rehabilitation for a patient with severe traumatic brain injury.	Wrong Population
M Guy	2021	Ethical Issues Linked to the Development of Telerehabilitation: a Qualitative Study.	Topic not aligned with the review scope
TE Strax	1994	Ethical issues of treating patients with aids in a rehabilitation setting	Wrong Population
A Hudon	2015	Ethical Issues Raised by Private Practice Physiotherapy Are More Diverse than First Meets the Eye: Recommendations from a Literature Review	Wrong study design
A Rochette	2014	Ethical issues relating to the inclusion of relatives as clients in the post-stroke rehabilitation process as perceived by patients, relatives and health professionals.	Wrong Population
SF Griech	2023	Ethical leadership in physical therapy: a developing construct that demands consideration.	Topic not aligned with the review scope
WA Hoffmann	2015	Ethical misconduct by registered physiotherapists in South Africa (2007-2013): A mixed methods approach.	Topic not aligned with the review scope
JD Banja	2013	Ethical perspectives on knowledge translation in rehabilitation.	Wrong Population
P Kyler -Hutchison	1988	Ethical reasoning and informed consent in occupational therapy.	Wrong Population
NN Sawi	2016	Ethical, Legal, and Medical Challenges When a Patient Refuses a Transfer From Rehabilitation to Acute Medical Services.	Wrong Population
B Fijalkowska	2012	Ethics and EBM in physiotherapy and in occupational therapy	Topic not aligned with the review scope
LL Swisher	2022	Ethics and Moral Agency for a Postpandemic Era: Beyond the Storm	Topic not aligned with the review scope
GR Scofield	1993	Ethics and rehabilitation medicine	Wrong Population
P Queruel	2014	Ethics and rehabilitation of the burn patient: How far to respect the principle of autonomy?	No Full Text
L Chiburis	1997	Ethics and rehabilitation of the patient with severe burns.	Wrong Population
L Pearson	1989	Ethics and rehabilitation--how to develop your ethical awareness.	Wrong Population
ED Metzeg	2002	Ethics corner: Cases from the Hebrew Rehabilitation Center for Aged - Restraint compliant	Wrong Population
ED Metzeg	2002	Ethics corner: cases from the Hebrew Rehabilitation Center for Aged--problematic proxies.	Wrong Population
ED Metzeg	2002	Ethics corner: cases from the Hebrew Rehabilitation Center for Aged--restraint complaint.	Wrong Population

ED Metzeg	2002	Ethics corner: cases from the Hebrew Rehabilitation Center for Aged.	Wrong Population
H Hardenbergh	1946	Ethics for the physical therapist, from the point of view of the medical practitioner.	Wrong Population
AR Haskins	2009	Ethics in geriatric rehabilitation	Wrong Population
WMM Levack	2009	Ethics in goal planning for rehabilitation: a utilitarian perspective.	Topic not aligned with the review scope
DR Falvo	2000	Ethics in rehabilitation education and research.	Wrong Population
G Yeo	1997	Ethics in rehabilitation with culturally diverse older adults	Wrong Population
CPF Pasquina	2015	Ethics in Rehabilitation: Access to Prosthetics and Quality Care Following Amputation.	Wrong Population
F Zaina	2016	Ethics in rehabilitation: challenges and opportunities to promote research.	Wrong Population
C Carpenter	2008	Ethics knowledge in physical therapy: a narrative review of the literature since 2000.	Wrong study design
B Kelly	2010	Ethics of involving children in health-related research: applying a decision-making framework to a clinical trial.	Wrong Population
F Ferrarello	2018	Ethics reporting practices in randomized controlled trials of physical therapy interventions after stroke.	Topic not aligned with the review scope
RB Purtilo	1978	Ethics teaching in allied health fields.	Topic not aligned with the review scope
JD Banja	1992	Ethics, fraud, and the misallocation of rehabilitation resources	Wrong Population
LV Monrouxe	2014	Even now it makes me angry': health care students' professionalism dilemma narratives.	Topic not aligned with the review scope
AC Kassberg	2008	Experiences of ethical dilemmas in rehabilitation: Swedish occupational therapists' perspectives.	Wrong Population
TJ Landon	2018	Exploring Rehabilitation Counseling Supervisors' Role in Promoting Counselor Development of Ethical Fluency.	Wrong Population
C Stiller	2000	Exploring the ethos of the physical therapy profession in the Unites States: Social, cultrual, and historical influences and their relationship to education	Topic not aligned with the review scope
HL Kordahl	2017	Facilitating awareness of philosophy of science, ethics and communication through manual skills training in undergraduate education.	Topic not aligned with the review scope
MD Hall	2015	Factors influencing physiotherapists' decisions to supervise physiotherapy students: Results from a Canadian national survey	Topic not aligned with the review scope
EJ Betan	1999	Fostering ethical willingness: Integrating emotional and contextual awareness with rational analysis.	Wrong Population
L Atanelov	2015	History of Physical Medicine and Rehabilitation and Its Ethical Dimensions.	Wrong Population
R. Hammond	2015	How physical therapists construct their professional identity	No Full Text
M Dillon	2023	How physiotherapists attend to the human aspects of care when working with people with low back pain: a thematic analysis.	Topic not aligned with the review scope
S Bashir	2018	Impact of social media on attitudes and professional growth of physical therapy students of foundation University, Islamabad, Pakistan	Topic not aligned with the review scope
MC Singleton	1987	Independent practice--on the horns of a dilemma. A special communication.	No Full Text
PJ Thomas	2005	Influence of academic qualifications, place of employment and prior research experience on physiotherapy research practice.	Topic not aligned with the review scope

E Marques-Sule	2022	Influence of Professional Values on Attitudes towards Professional Ethics in Future Physical Therapy Professionals.	Topic not aligned with the review scope
AL Caplan	1988	Informed consent and provider-patient relationships in rehabilitation medicine.	Wrong Population
JR Carlisle	2002	Informed consent in physical medicine and rehabilitation. The physician/patient relationship--the doctor as a fiduciary.	Wrong Population
VM Tarvydas	1996	Interdisciplinary team member perceptions of ethical issues in traumatic brain injury rehabilitation.	Topic not aligned with the review scope
JD Strawbridge	2014	Interprofessional ethics and professionalism debates: findings from a study involving physiotherapy and pharmacy students.	Topic not aligned with the review scope
GM Jensen	2010	Interprofessional ethics in rehabilitation: the dreamcatcher journey.	Topic not aligned with the review scope
R Garlikov	2008	Introduction to perspectives on ethical issues and dilemmas in the treatment of patients with spinal cord injury	Wrong Population
K Mostert	2012	Involvement in, and views on, social responsibility of physiotherapists from a South African province: A cross-sectional survey	Topic not aligned with the review scope
M Izquierdo	2016	Is It Ethical Not to Precribe Physical Activity for the Elderly Frail?	Wrong Population
EE Madsen	2016	Is therapeutic judgement influenced by the patient's socio-economic status? A factorial vignette survey.	Wrong Population
RB Purtilo	1981	Justice in the distribution of health care resources. The position of physical therapists, physiatrists, and rehabilitation nurses.	No Full Text
I Canjuga	2024	Justice sensitivity among nurses and physiotherapists in a Croatian rehabilitation hospital.	Topic not aligned with the review scope
KS Aderibigbe	2019	Knowledge and practice of informed consent by physiotherapists and therapy assistants in KwaZulu-Natal Province, South Africa.	Topic not aligned with the review scope
A Sharp	2021	Lapses in Professional Behavior Identified by Students of Physical Therapy.	Topic not aligned with the review scope
A Hudon	2015	Legal and ethical challenges in the private rehabilitation sector.	No Full Text
G Gard	2005	Life-views and ethical viewpoints among physiotherapy students in Sweden and Turkey - A comparative study	Topic not aligned with the review scope
I Edwards	2011	Living a moral professional life amidst uncertainty: Ethics for an Afghan physical therapy curriculum	Topic not aligned with the review scope
RB Purtilo	1995	Managed care: ethical issues for the rehabilitation professions.	No Full Text
AS Nica	2019	Management and ethics issues in rehabilitation and balneoclimatology	Topic not aligned with the review scope
HM Larin	2009	Measuring moral judgement in physical therapy students from different cultures: a dilemma	Topic not aligned with the review scope
J Blackmer	2002	Medical-ethics teaching in Canadian physical medicine and rehabilitation residency training programs.	Wrong Population
J Sim	1989	Methodology and Morality in Physiotherapy Research	Topic not aligned with the review scope
MS Baliga	2024	Moral Distress and Dilemmas Faced by Health Care Workers During Screening, Treating, and Rehabilitating Women with Gynecological Cancer: A Narrative Review from a Bioethics Consortium	Wrong Population
C Carpenter	2010	Moral distress in physical therapy practice	Topic not aligned with the review scope
D Mukherjee	2009	Moral distress in rehabilitation professionals: results from a hospital ethics survey	Topic not aligned with the review scope

MM Green	2017	Moral Distress in Rehabilitation.	Wrong Population
S WSisola	2000	Moral reasoning as a predictor of clinical practice: the development of physical therapy students across the professional curriculum.	Topic not aligned with the review scope
T Jorgensen-Smith	2021	Multicultural ethics in rehabilitation services	Wrong Population
E Durocher	2010	Navigating ethical discharge planning: A case study in older adult rehabilitation.	Wrong Population
K Naido	2020	Navigating without a Compass: How Culturally and Linguistically Diverse Physical Therapist Students Persist in Higher Education.	Topic not aligned with the review scope
AJ Porcino	2014	Negotiating Consent: Exploring Ethical Issues when Therapeutic Massage Bodywork Practitioners Are Trained in Multiple Therapies.	Topic not aligned with the review scope
JA Balogun	2017	Nigerian physiotherapists' knowledge and attributes of professionalism	Topic not aligned with the review scope
M Frilund	2021	Nordic forum for ethics in physiotherapy	No Full Text
MS Hall	2022	Patient and practitioner perspectives of psychological need support in physical therapy.	Topic not aligned with the review scope
I Milinkovic	2014	PATIENT'S RIGHT TO INFORMED CONSENT IN REPUBLIC SRPSKA: LEGAL AND ETHICAL ASPECTS (WITH SPECIAL REFERENCE TO PHYSICAL REHABILITATION).	No Full Text
AK Wagner	2003	Peer review: issues in physical medicine and rehabilitation.	Topic not aligned with the review scope
B Greenfield	2009	Phenomenology: an alternative ethics in rehabilitation.	Wrong study design
DL Lowe	2015	Physical Therapist Student Experiences With Ethical and Legal Violations During Clinical Rotations: Reporting and Barriers to Reporting.	Topic not aligned with the review scope
EL Geddes	2009	Physical therapist students as moral agents during clinical experiences	Topic not aligned with the review scope
A Mansbach	2010	Physical therapists' awareness and reporting of peer misconduct: A survey	No Full Text
R Cantu	2019	Physical therapists' perception of workplace ethics in an evolving health-care delivery environment: a cross-sectional survey	Topic not aligned with the review scope
BA Arcos	2021	Physical therapy bioethical reflections on disabled persons.	Topic not aligned with the review scope
P Galley	1977	Physiotherapists as first contact practitioners: new challenges and responsibilities in Australia	Topic not aligned with the review scope
L Mari	2019	Physiotherapists need to engage in a person-centred ethics for better health	Wrong study design
MR Elkins	2020	Physiotherapists should consider joining an ethics review committee.	Topic not aligned with the review scope
A Sillero	2023	Physiotherapists' Ethical Climate and Work Satisfaction: A STROBE-Compliant Cross-Sectional Study.	Topic not aligned with the review scope
N Inbar	2024	Physiotherapists' moral distress: Mixed-method study reveals new insights.	Topic not aligned with the review scope
B McPhee	1977	Physiotherapy - open minds?	Topic not aligned with the review scope
D Malcolm	2014	Practical responses to confidentiality dilemmas in elite sport medicine.	Topic not aligned with the review scope

J Ròzynska	2012	Principles for research in physiotherapy involving human subjects	Topic not aligned with the review scope
X Wang	2020	Professionalism dilemmas experienced by health professions students: a cross-sectional study.	Topic not aligned with the review scope
K Gagnon	2015	Professionalism in a digital age: opportunities and considerations for using social media in health care.	Topic not aligned with the review scope
A Townsend	2010	Qualitative research ethics: enhancing evidence-based practice in physical therapy.	Topic not aligned with the review scope
RH Meier	1988	Recent developments in rehabilitation giving rise to important new (and old) ethical issues and concerns.	Wrong Population
GM Jensen	2005	Reflection on the teaching of ethics in physical therapist education: integrating cases, theory, and learning.	Topic not aligned with the review scope
K Reidy	1991	Refusing treatment during rehabilitation. A model for conflict resolution	Wrong Population
RB Purtilo	1991	Rehabilitation and technology: ethical considerations.	No Full Text
FJ Lane	2012	Rehabilitation Counselors' Perceptions of Ethical Workplace Culture and the Influence on Ethical Behavior.	Wrong Population
A De Martini	2011	Rehabilitation, ethics and technique].	Wrong Population
SA Salladay	1996	Rehabilitation, ethics, and managed care.	No Full Text
LD Henley	2006	Reporting ethical protections in physical therapy research.	Wrong study design
S Sabapathy	2009	Reporting of ethical issues in Indian Physiotherapy journals	Wrong study design
CJ Kim	2017	Research and publication ethics of the Journal of Exercise Rehabilitation	Wrong Population
EJ Phipps	2000	Research Ethics in Head Trauma Rehabilitation	Wrong Population
J Sim	1998	Respect for autonomy: issues in neurological rehabilitation.	Wrong Population
RT Hare-Mustin	1979	Rights of clients, responsibilities of therapists.	Wrong Population
DK Anderson	2013	Self-assessment of professionalism in physical therapy education.	Topic not aligned with the review scope
I Cooper	2008	Sexual boundaries between physiotherapists and patients are not perceived clearly: an observational study	Topic not aligned with the review scope
RC Hollyday	2007	Should patients participate in clinical decision making? An optimised balance block design controlled study of goal setting in a rehabilitation unit.	Wrong Population
AF Viero Badarò	2011	Sociodemographic and professional profile of physical therapists and origin of their conceptions of ethics.	Topic not aligned with the review scope
No authors listed	1997	Standards of practice for physical therapy and the accompanying criteria. The American Physical Therapy Association.	No Full Text
RB Purtilo	1979	Structure of ethics teaching in physical therapy: a survey.	No Full Text
AS Ragusea	2003	Suggestions for the ethical practice of online psychotherapy.	Wrong Population
A Hudon	2018	Supporting ethics educators in Canadian occupational therapy and physical therapy programs: A national interprofessional knowledge exchange project.	Topic not aligned with the review scope
A Sivasankari	2021	Survey on parental attitude towards ethical considerations of involving their children in physiotherapy care	Topic not aligned with the review scope

I Cooper	2013	Targeted education on the topic of professional boundaries does not change student physiotherapists' opinions or their responses to a series of ethical scenarios.	Topic not aligned with the review scope
HL Triezenberg	1997	Teaching ethics in physical therapy education: a Delphi study.	No Full Text
P O' Neill	1998	Teaching ethics: The utility of the CPA code.	Wrong Population
A Bialocerkowsk i	2011	Teaching physiotherapy skills in culturally-diverse classes.	Topic not aligned with the review scope
S Murphy	2020	Teaching professionalism: some features in Canadian physiotherapy programs.	Topic not aligned with the review scope
LD Gilbert	1973	The changing work ethic and rehabilitation.	Wrong Population
VM Tarvydas	2000	The Code of Ethics for Professional Rehabilitation Counselors: what we have and what we need.	Wrong Population
R Hammond	2016	The construction of professional identity by physiotherapists: a qualitative study	Topic not aligned with the review scope
S Kumar	2010	The ethics of evidence implementation in health care.	Topic not aligned with the review scope
A Caplan	1997	The ethics of gatekeeping in rehabilitation medicine	Wrong Population
J Sim	1994	The ethics of single-system (n = 1) research	No Full Text
ID Da Silva	2011	The humanization and the formation of the professional in physiotherapy].	Topic not aligned with the review scope
BH Greenfield	2006	The meaning of caring in five experienced physical therapists.	Topic not aligned with the review scope
EL Ramsden	1975	The patient's right to know. Implications for interpersonal communication processes.	No Full Text
ST Wegener	1996	The rehabilitation ethic and ethics.	Wrong Population
T Dawn	2019	The Relationship Between Completion of Postprofessional Orthopedic Manual Physical Therapy Education and Core Values of Professionalism.	Topic not aligned with the review scope
S Byrd	2011	The right not to hear: the ethics of parental refusal of hearing rehabilitation.	Wrong Population
RT Guenther	1996	The role of an ethics committee in a rehabilitation setting.	Topic not aligned with the review scope
JF Haas	1993	The role of ethics in rehabilitation medicine. Introduction to a series.	Wrong Population
JK Silver	2020	The Vital Role of Professionalism in Physical Medicine and Rehabilitation.	Wrong Population
K Dholakia	2023	Transforming Society Through Critical Service-Learning: A Position for a Justice-Based Approach to Experiential Learning in Physical Therapy Education	No Full Text
R Barnitt	1994	Truth Telling in Occupational Therapy and Physiotherapy.	Wrong Population
K Dholakia	2021	Uncovering Ethical Dilemmas in International Service- Learning: A Grounded Theory.	Topic not aligned with the review scope
M Hughes	2009	Use of deconstructed cases in physical therapy ethics education: an assessment of student learning.	Topic not aligned with the review scope
A Hudon	2015	What are the ethical issues faced by physiotherapists working in private practice? Results of a literature review	No Full Text

D Rasoal	2016	What healthcare teams find ethically difficult.	Wrong Population
A Hudon	2014	What place for ethics? An overview of ethics teaching in occupational therapy and physiotherapy programs in Canada.	Topic not aligned with the review scope
JD Banja	1985	Whistleblowing in physical therapy.	No Full Text
MJ Naylor	2022	You've broken the patient": Physiotherapists' lived experience of incivility within the healthcare team - An Interpretative Phenomenological Analysis.	Topic not aligned with the review scope
Greenfield B.	2012	Technology in Rehabilitation: Ethical and Curricular Implications for Physical Therapist Education	No Full Text
Greenfield B.	2009	The Role of Ethical Theory in Ethical Education for Physical Therapist Students	No Full Text

Supplementary Material 3 – Detailed List of Included Studies

Author	Year	Country	Title	Ethical Inquiry Type	Study design	Domains of physical therapy
			**Ethical theory**			
Bruckner J.	1987	USA	Physical therapists as double agents. Ethical dilemmas of divided loyalties.	Normative	Case study	Generic
Delany C.	2010	Australia	Closing the gap between ethics knowledge and practice through active engagement: an applied model of physical therapy ethics.	Normative	Ethical Analysis	Generic
Delany C.	2005	Australia	Respecting patient autonomy and obtaining their informed consent: ethical theory-missing in action	Normative	Ethical Analysis	Generic
Drolet M.	2015	Canada	Theoretical frameworks used to discuss ethical issues in private physiotherapy practice and proposal of a new ethical tool	Normative	Review	Generic
Frilund M.	2021	Norway	The Ethical Standpoints of Rehabilitation in the Nordic Countries- A Theoretical Study About Caring Sciences and Rehabilitation.	Normative	Ethical Analysis	Generic
Gorman-Badar D.	2024	USA	Particularizing an Internal Morality of Physical Therapy	Normative	Ethical Analysis	Generic
Greenfield B.	2010	USA	Beyond a code of ethics: phenomenological ethics for everyday practice.	Normative	Editorial	Generic
Greenfield B.	2009	USA	Phenomenology - An Alternative Ethics in Rehabilitation	Normative	Editorial	Generic
Levack M.	2009	New Zealand	Ethics in goal planning for rehabilitation: a utilitarian perspective	Normative	Ethical Analysis	Generic
McPherson GW	2003	Canada	Rehabilitation: disability ethics versus Peter Singer.	Normative	Ethical Analysis	Generic
Purtilo R.	1975	USA	Reading “Physical Therapy” from an Ethics Perspective	Normative	Editorial	Generic
Sviland R.	2022	Norway	Løgstrup’s thinking: a contribution to ethics in physiotherapy	Normative	Ethical Analysis	Generic
Key Message: Critiques of deontological ethics favor contextual models (e.g., the "active engagement model"), integrating patients’ social identities and relational dynamics. Theoretical tensions arise between utilitarianism (e.g., goal planning in resource-limited contexts) and ethics affirming the intrinsic value of persons with disabilities, advocating hybrid frameworks for rehabilitation.						
			**Ethical reasoning**			
Araújo LZS	2023	Mexico	Bioethical aspects of physiotherapy in the intensive care unit	Normative	Ethical Analysis	Intensive Care
Banja JD	1993	USA	Ethical issues in treating pediatric rehabilitation patients.	Normative	Case study	Pediatric
Barnitt R.	1997	United Kingdom	Ethical reasoning in physical therapy and occupational therapy.	Empirical	Qualitative study	Generic
Chigbo NN	2015	Nigeria	Ethics of physiotherapy practice in terminally ill patients in a developing country, Nigeria	Normative	Review	Palliative care
Clarke S.	2016	Canada	Ethics and Community-Based Rehabilitation: Eight Ethical Questions from a Review of the Literature.	Normative	Review	Generic
Clawson A.	1994	USA	The relationship between clinical decision making and ethical decision making	Normative	Ethical Analysis	Generic
Delany C.	2015	Australia	An ethical approach to health promotion in physiotherapy practice.	Normative	Case study	Generic
Delany C.	2019	Australia	How physiotherapists perceive, interpret, and respond to the ethical dimensions of practice: A qualitative study.	Empirical	Qualitative study	Generic
Delany C.	2012	Australia	The role of clinical ethics consultations for physical therapy practice	Normative	Ethical Analysis	Generic

Ditwiler R.	2024	USA	Definitely a Dark Time:" professional and ethical issues in post-acute care physical therapy during the COVID-19 pandemic.	Empirical	Qualitative study	Infectious diseases
Ditwiler R.	2022	USA	Doing things you never imagined: Professional and ethical issues in the U.S. outpatient physical therapy setting during the COVID-19 pandemic.	Empirical	Qualitative study	Infectious diseases
Edwards I.	2005	Australia	Ethical reasoning as a clinical-reasoning strategy in physiotherapy	Normative	Ethical Analysis	Generic
Finch E.	2005	Canada	Ethically based clinical decision-making in physical therapy: process and issues.	Empirical	Qualitative study	Generic
Galley P.	1975	Australia	Ethical principles and patient referral	Normative	Editorial	Generic
Greenfield B.	2012	USA	Ethical issues in sports medicine: a review and justification for ethical decision making and reasoning.	Normative	Ethical Analysis	Sport
Guccione AA	1980	USA	Ethical issues in physical therapy practice. A survey of physical therapists in New England.	Empirical	Survey	Generic
Haskins A.	2009	USA	Ethical issues in geriatric rehabilitation: A culturally competent approach	Normative	Ethical Analysis	Geriatric
Hudon A.	2019	Canada	Tensions Living Out Professional Values for Physical Therapists Treating Injured Workers	Empirical	Qualitative study	Generic
Kulju K.	2020	Finland	Self-evaluated ethical competence of a practicing physiotherapist: a national study in Finland	Empirical	Observational study	Generic
Malarvizhi D.	2021	India	Ethical issues at the interface of physiotherapy care and research practice in pediatric oncology-descriptive study	Empirical	Observational study	Pediatric
Nalette E.	2010	USA	Constrained physical therapist practice: an ethical case analysis of recommending discharge placement from the acute care setting.	Normative	Case study	Generic
Naudé A	2017	South Africa	Measuring Instrument for Ethical Sensitivity in the Therapeutic Sciences.	Empirical	Mixed Methods	Generic
Poulis I.	2007	USA	Bioethics and physiotherapy	Normative	Editorial	Generic
Praestegaard J.	2013	Sweden	Practicing physiotherapy in Danish private practice: an ethical perspective.	Empirical	Qualitative study	Generic
Praestegaard J.	2011	Sweden	The perceptions of danish physiotherapists on the ethical issues related to the physiotherapist-patient relationship during the first session: a phenomenological approach	Empirical	Qualitative study	Generic
Purtilo R.	1974	USA	Understanding ethical issues. The physical therapist as ethicist	Normative	Ethical Analysis	Generic
Riendeau C.	2015	Canada	An investigation of how university sports team athletic therapists and physical therapists experience ethical issues	Empirical	Qualitative study	Sport
Scheirton L.	2007	USA	Error and patient safety: Ethical analysis of cases in occupational and physical therapy practice	Normative	Case study	Generic
Sim J.	1991	United Kingdom	An Ethical Analysis of Physical Therapists' Duty to Treat Persons Who Have AIDS: Homosexual Patients as a Test Case	Normative	Editorial	Infectious diseases
Sousa J.	2021	Portugal	Ageing and ethical challenges in physiotherapy: application of the RIPS model in ethical decision-making	Normative	Case study	Geriatric
Sturm A.	2023	Austria	Views of physiotherapists on factors that play a role in ethical decision-making: an international online survey study.	Empirical	Survey	Generic
Swisher LL	2010	USA	Moral reasoning among physical therapists: results of the Defining Issues Test.	Empirical	Survey	Generic
Uddin T.	2022	Pakistan	Ethical issues and dilemmas in spinal cord injury rehabilitation in the developing world: a mixed-method study	Empirical	Mixed Methods	Neurologic

Key Message: Ethical reasoning in physical therapy is often guided by pragmatic intuitions and contextual models (e.g., the RIPS framework). However, tensions between ethical principles and external pressures (e.g., productivity targets, medical hierarchies) lead to "moral compromises." Studies in sports medicine and post-COVID rehabilitation highlight the need for structured tools (e.g., ethical consultations) to balance patient autonomy, distributive justice, and systemic constraints.

			**Ethical reasoning and Education**			
Aguilar - Rodriguez	2021	Spain	Physiotherapy Students' Experiences about Ethical Situations Encountered in Clinical Practices	Empirical	Qualitative study	Generic
Arnal-Gómez A.	2022	Spain	Professional values and perception of knowledge regarding professional ethics in physical therapy students: A STROBE compliant cross-sectional study.	Empirical	Cross-sectional	Generic
Caenazzo L.	2020	Italy	Teaching ethics and professionalism in rehabilitation: an empirical research on active learning with university rehabilitation students.	Empirical	Observational study	Generic
Carey JR.	2019	USA	Academic Ethos in Physical Therapy Education	Normative	Ethical Analysis	Generic
Deglio Alves F.	2008	Brasil	Bioethical education in physical therapy undergraduate course	Empirical	Survey	Generic
Dieruf K.	2004	USA	Ethical decision-making by students in physical and occupational therapy	Empirical	Longitudinal study	Generic
Edwards I.	2024	Australia	The development of moral judgment and organization of ethical knowledge in final year physical therapy students.	Empirical	Longitudinal study	Generic
Geddes EL	2008	Canada	Does moral judgement improve in occupational therapy and physiotherapy students over the course of their pre-licensure training?	Empirical	Longitudinal study	Generic
Geddes EL	2009	Canada	Ethical issues identified by physical therapy students during clinical placements	Normative	Review	Generic
Howard B.	2020	USA	Comparing Moral Reasoning Across Graduate Occupational and Physical Therapy Students and Practitioners...2020 AOTA Annual Conference & Expo.	Empirical	Cross-sectional	Generic
Jiandani MP	2022	India	Teaching bioethics needs more than just a module: A comparison of knowledge and attitude of bioethics principles in 1st- and 3rd-year physiotherapy undergraduates.	Empirical	Survey	Generic
Lowe D.	2014	USA	Physical Therapist Student Experiences With Ethical and Legal Violations During Clinical Rotations: Reporting and Barriers to Reporting	Empirical	Survey	Generic
Macpherson I	2021	Spain	Analysis in the ethical decision-making of dental, nurse and physiotherapist students, through case-based learning	Empirical	Mixed Methods	Generic
Romanello M.	2000	USA	The "Ethic of Care" in Physical Therapy Practice and Education: Challenges and Opportunities	Normative	Editorial	Generic
Sturm A.	2024	Austria	Western ideals and global realities–physiotherapists' views on factors that play a role in ethical decision-making: an international qualitative analysis	Empirical	Qualitative study	Generic
Sutkowi-Hemstreet	2025	USA	Justice, Equity, Diversity, and Inclusion–Related Curricular Elements in Entry-Level Physical Therapist Education: A Delphi Study	Empirical	Delphy	Generic
Swisher LL	2012	USA	Evaluating moral reasoning outcomes in physical therapy ethics education: stage, schema, phase, and type.	Empirical	Longitudinal study	Generic
Triezenberg H.	2001	USA	The Use of Narrative in an Applied Ethics Course for Physical Therapist Students	Empirical	Survey	Generic
Triezenberg H.	2000	USA	Beyond the Code of Ethics: Educating Physical Therapists for Their Role as Moral Agents	Normative	Ethical Analysis	Generic
Key Message: Effective ethics education requires experiential approaches: case-based learning (CBL) enhances students' moral sensitivity, while traditional curricula show limited outcomes. Disparities between students and professionals (e.g., clinicians' advanced ethical reasoning) underscore the importance of clinical placements, mentorship, and critical reflection to integrate theory and practice.						
			**Ethical Perception**			
Barnitt R.	1998	United Kingdom	Ethical dilemmas in occupational therapy and physical therapy: a survey of practitioners in the UK National Health Service.	Empirical	Survey	Generic
Berg-Poppe P.	2019	USA	The impact of an evolving profession on the frequency and perceived difficulty of ethical encounters among physical therapists in the clinic.	Empirical	Survey	Generic
Ditwiler R.	2021	USA	Professional and Ethical Issues in United States Acute Care Physical Therapists Treating Patients With COVID-19: Stress, Walls, and Uncertainty.	Empirical	Qualitative study	Infectious diseases

Fryer C.	2021	Australia	Scarcity of resources and inequity in access are frequently reported ethical issues for physiotherapists internationally: an observational study.	Empirical	Observational study	Generic
Kulju K.	2013	Finland	Ethical problems and moral sensitivity in physiotherapy	Empirical	Survey	Generic
Moreno - Segura N.	2023	Spain	Physical Therapists' Ethical and Moral Sensitivity: A STROBE-Compliant Cross-Sectional Study with a Special Focus on Gender Differences.	Empirical	Cross-sectional	Generic
Nyante GG	2020	Ghana	Patterns of ethical issues and decision-making challenges in clinical practice among Ghanaian physiotherapists.	Empirical	Cross-sectional	Generic
Praestegaard J.	2013	Sweden	Ethical issues in physiotherapy--reflected from the perspective of physiotherapists in private practice.	Empirical	Qualitative study	Generic
Skiba D.	2023	Poland	Moral values in the work of a physiotherapist	Empirical	Survey	Generic
Sohail M.	2021	Pakistan	Knowledge, Interest and Perception of Academic Physiotherapists with Regard to Professional Ethics	Empirical	Cross-sectional	Generic
Sturm A.	2022	Austria	Almost) 50 shades of an ethical situation - international physiotherapists' experiences of everyday ethics: a qualitative analysis.	Empirical	Qualitative study	Generic
Tarvydas VM	1996	USA	Interdisciplinary team member perceptions of ethical issues in traumatic brain injury rehabilitation	Empirical	Survey	Neurologic
Triezenberg HL	1996	USA	The identification of ethical issues in physical therapy practice.	Empirical	Delphi	Generic
Key Message: Perceptions of ethical dilemmas vary culturally: private-sector physical therapists (e.g., Sweden, USA) prioritize patient autonomy, while public-sector practitioners (e.g., Canada, Australia) face tensions between equity and institutional mandates. Gender differences (e.g., higher ethical sensitivity in women) and training gaps demand flexible guidelines and context-specific continuing education.						
**Ethics of care relationship**						
†	2019	Sweden	Ethics and sexual health: Exploration of the ethical code of conduct for physiotherapists concerning sexual health in clinical practice.	Normative	Ethical Analysis	Generic
Bellner AL.	1999	Sweden	Senses of responsibility. A challenge for occupational and physical therapists in the context of ongoing professionalization.	Normative	Ethical Analysis	Generic
Bettini-Pereira RA	2014	Brasil	Reflexões bioéticas em fisioterapia sobre a pessoa com deficiência.	Normative	Review	Generic
Cardol M.	2002	Netherlands	On autonomy and participation in rehabilitation.	Normative	Review	Generic
Copnell G.	2018	United Kingdom	Informed consent in physiotherapy practice: it is not what is said but how it is said	Normative	Ethical Analysis	Generic
Coy JA	1989	USA	Autonomy-Based Informed Consent: Ethical Implications for Patient Noncompliance	Normative	Editorial	Generic
Dahl-Michelsen T.	2019	Norway	Approaching intimacy, sexuality and ethics in the professional training of physiotherapy students in Norway	Empirical	Qualitative study	Generic
Delany C.	2007	Australia	In private practice, informed consent is interpreted as providing explanations rather than offering choices: a qualitative study.	Empirical	Qualitative study	Generic
Długołęcka A.	2024	Poland	Ethics of a Physiotherapist: Touch, Corporeality, Intimacy—Based on the Experience of Elderly Patients	Empirical	Qualitative study	Geriatric
Haswell K.	1996	Australia	Informed choice and consent for cervical spine manipulation	Normative	Editorial	Muscolo-skeletal
Lees AB	2012	New Zealand	To tell or not to tell? Physiotherapy students' responses to breaking patient confidentiality.	Empirical	Longitudinal study	Generic
Mármol-López M.	2023	Spain	Physiotherapists' ethical behavior in professional practice: a qualitative study.	Empirical	Qualitative study	Generic
Okezue OC	2023	Nigeria	Patient involvement in medical decisions: a survey of shared decision making during physical therapy consultations.	Empirical	Survey	Generic

Purtilo R.	1984	USA	Applying the principles of informed consent to patient care. Legal and ethical considerations for physical therapy.	Normative	Editorial	Generic
Roman N.	2019	Romania	Ethical considerations about informed consent in physiotherapy in Romania.	Empirical	Cross-sectional	Generic
Key Message: Physical therapy poses unique dilemmas tied to physical touch, power asymmetry, and informed consent. Studies on elderly or vulnerable patients (e.g., Poland, 2024) reveal consent is often interpreted as technical explanation rather than shared decision-making. Ethics training must emphasize empathetic communication, professional boundaries, and managing moral distress from organizational constraints.						
**Justice and equity in clinical ethics**						
Cantu R.	2019	USA	Physical Therapists' Ethical Dilemmas in Treatment, Coding, and Billing for Rehabilitation Services in Skilled Nursing Facilities: A Mixed-Method Pilot Study	Empirical	Mixed Methods	Generic
Dholakia K.	2023	USA	Transforming Society Through Critical Service-Learning: A Position for a Justice-Based Approach to Experiential Learning in Physical Therapy Education	Normative	Ethical Analysis	Generic
Edwards I.	2011	Australia	New Perspectives on the Theory of Justice: Implications for Physical Therapy Ethics and Clinical Practice	Normative	Ethical Analysis	Generic
Hunt M.	2013	Canada	A patient-centered care ethics analysis model for rehabilitation	Normative	Ethical Analysis	Generic
Laliberté M.	2017	Canada	Ethical Challenges for Patient Access to Physical Therapy: Views of Staff Members from Three Publicly-Funded Outpatient Physical Therapy Departments.	Empirical	Qualitative study	Generic
Palad Y.	2024	Philippine s	Physical Therapists' Social Responsibility in the Philippines Entails Adopting a Societal Practice Framework: A Qualitative Study.	Empirical	Qualitative study	Generic
Purtilo R.	1982	USA	Justice in the Distribution of Health Care Resources: The Position of Physical Therapists in the United States and Sweden	Empirical	Survey	Generic
Purtilo R.	1992	USA	Whom to Treat First, and How Much is Enough?: Ethical Dilemmas that Physical Therapists Confront as They Compare Individual Patients' Needs for Treatment	Normative	Ethical Analysis	Generic
Key Message: In public or resource-limited settings (e.g., Philippines, Ghana), physical therapists face dilemmas over resource allocation and treatment prioritization. Studies propose needs-based justice models (e.g., Sweden) and social responsibility (e.g., advocacy for rural communities), emphasizing education's role in preparing professionals for systemic challenges.						
**Codes of ethics**						
Anderson L.	2013	New Zealand	Engaging the professional community: rewriting a code of ethics for NZ physiotherapists.	Empirical	Qualitative study	Professional Ethics
Linker B.	2005	USA	The business of ethics: gender, medicine, and the professional codification of the American Physiotherapy Association, 1918-1935.	Normative	Ethical Analysis	Professional Ethics
Mohamadi M.	2024	Iran	Assessing Physiotherapists' Knowledge of Professional Ethics Codes in Shiraz: A Cross-Sectional Study.	Empirical	Cross-sectional	Professional Ethics
Mohamadi M.	2022	Iran	Proposing a set of ethical guidelines for Iranian physiotherapists: results of a modified Delphi technique	Normative	Delphi	Professional Ethics
Pezdek K.	2023	Poland	The Ethical Code of Conduct for Physiotherapists—An Axiological Analysis	Normative	Ethical Analysis	Professional Ethics
Purtilo R.	1987	USA	Codes of ethics in physiotherapy: A retrospective view and look ahead	Normative	Editorial	Professional Ethics
Swisher LL	2010	USA	The Revised APTA Code of Ethics for the Physical Therapist and Standards of Ethical Conduct for the Physical Therapist Assistant: Theory, Purpose, Process, and Significance	Normative	Review	Professional Ethics
Tamar J.	2014	Israel	Assimilation of the Patient Rights Law and Code of Ethics into Israeli Physical Therapy Services.	Empirical	Qualitative study	Professional Ethics
Key Message: Studies reveal that physical therapy codes of ethics (e.g., the APA Code, 1918–1935) are dynamic tools historically used to legitimize the profession. However, discrepancies persist between formal principles and daily practice. Structured training and contextual internalization of codes are critical to bridge implementation gaps, especially in resource-limited settings or under institutional pressures (e.g., economic priorities vs. equity)						
